# Acceptability, feasibility, and effectiveness of internet-based cognitive behavior therapy for obsessive–compulsive disorder (OCD-NET): a naturalistic pilot trial during the COVID-19 pandemic in a psychiatric outpatient department in Germany

**DOI:** 10.1186/s12888-025-06519-7

**Published:** 2025-01-30

**Authors:** Elisabeth Kohls, Sabrina Baldofski, Julia Scholl, Oskar Flygare, Lina Lundström, Ursula Beyrich-Kolbus, Marc Steinbrecher, Christian Rück, Christine Rummel-Kluge

**Affiliations:** 1 Department of Psychiatry and Psychotherapy, Medical Faculty, Leipzig University, Leipzig, Germany; 2 Department of Psychiatry and Psychotherapy, University of Leipzig Medical Center, Leipzig University, Leipzig, Germany; 3 Centre for Psychiatry Research, Department of Clinical Neuroscience, Karolinska Institutet, & Stockholm Health Care Services, Region Stockholm, Stockholm, Sweden; 4 Klinik Und Poliklinik Für Psychiatrie Und Psychotherapie, Semmelweisstr. 10, Haus 13, Leipzig, 04103 Germany

**Keywords:** Internet, ICBT, Cognitive-behavioral therapy, Treatment, Psychiatric patients, OCD, COVID-19

## Abstract

**Background:**

Cognitive behavior therapy (CBT) is the gold-standard treatment for obsessive–compulsive disorder (OCD). However, access to CBT and specialized treatments is often limited. This pilot study describes the implementation of a guided Internet-Based CBT program (ICBT) for individuals seeking treatment for OCD in a psychiatric outpatient department in Leipzig, Germany, during the COVID-19 pandemic. The aim of the study was to investigate the acceptability, feasibility, and effectiveness of the ICBT program for OCD.

**Methods:**

In an open, naturalistic pilot trial, *N* = 57 patients with OCD received a 10-week ICBT program (called “OCD-NET”). It consisted of 10 different modules covering psychoeducation, cognitive restructuring, exposure with response prevention, and overall therapist support and guidance through the program. The primary outcome was feasibility and acceptance of the OCD-NET program assessed via recruitment and retention rate, adherence and user satisfaction. Secondary outcomes were OCD symptoms at the end of treatment, assessed using the self-report Obsessive Compulsive Inventory – Revised (OCI-R) and self-rated measures of depressive symptoms, quality of life, self-efficacy, and psychological distress. Additionally, treatment credibility, working alliance, and satisfaction were assessed.

**Results:**

On average, participants completed 6.30 (*SD* = 3.21) modules, and *n* = 19 (33.9%) participants completed all 10 modules of the program. Overall, *n* = 45 (78.9%) were treatment completers (minimum 4 modules completed), *n* = 11 (19.3%) were non-completers, and *n* = 1 (1.8%) was a dropout. Satisfaction with the program was high, with a majority of participants indicating that they would recommend it to others (*n* = 56, 98.2%) and that it provided the support they needed (*n* = 49, 86.0%).Mixed-effect models showed a significant reduction in OCD symptoms (OCI-R), with large within-group effect sizes in both intention-to-treat (ITT) and completer analyses. In ITT analyses, the OCI-R decreased significantly with a within-group effect size of *d* = 1.13 (95% CI 0.88 – 1.38). At post-treatment, *n* = 17 (29.8%) participants showed a treatment response on the OCI-R (≥ 40% reduction). The treatment also resulted in statistically significant improvements in depressive symptoms (*d* = 0.90 [0.65; 1.15]) and self-efficacy (*d* = -0.27 [-0.53; -0.00]). No significant differences were observed in quality of life (WHOQOL-BREF) or psychological distress (Mini-SCL GSI) scores between baseline and post-treatment, in either the ITT or completer analyses.

**Conclusions:**

The OCD-NET program is overall highly acceptable and appears to meet patients’ needs in routine care, even under pandemic constraints. ICBT with therapist guidance significantly reduces OCD and depressive symptoms in real world settings. The results also suggest that this ICBT program could be integrated into routine psychiatric outpatient treatments. However, future research should investigate how upscaling and sustainable implementation could be effectively achieved.

**Trial registration:**

German Clinical Trials register (DRKS): DRKS00021706, registration date: 15.05.2020.

**Supplementary Information:**

The online version contains supplementary material available at 10.1186/s12888-025-06519-7.

## Introduction

Obsessive–compulsive disorder (OCD) has an estimated lifetime prevalence of 2–3% [1]. OCD typically has an onset between 18 and 25 years and follows a chronic course if left untreated [2–6]. Individuals affected by OCD experience intrusive and unwanted thoughts (obsessions) and repetitive, time-consuming behaviors (compulsions) [7], which significantly reduce their quality of life and social functioning [8, 9]. The time from onset to diagnosis is remarkably long (12 years on average), as is the time from diagnosis to treatment (1.45 years on average) [6].

A number of interventions are effective in managing OCD, including pharmacological and psychological treatments [10]. Cognitive-behavioral therapy (CBT), particularly exposure and response prevention (ERP), is recommended as the first-line psychological treatment for OCD [11, 12]. Access to specialized treatment is limited due to several factors and barriers [13]. Some patients do not seek professional help due to barriers such as the cost of treatment, lack of insurance coverage, stigma associated with the disorder [14], or doubts about its effectiveness [15, 16]. Another significant barrier is the shame and expected inconveniences associated with the treatment [17]. Furthermore, specialized CBT treatments for OCD are limited within the German healthcare system, similarly to other European countries [13, 18]. The COVID-19 pandemic required mental health services around the world to adapt quickly to new restrictions and regulations [19]. Additionally, people with OCD were particularly affected by the early stages of the pandemic and the subsequent pandemic containment measures and governmental regulations, which also hindered access to treatment [20–24].

In recent years, internet-delivered cognitive-behavioral therapy (ICBT) has been established to address these issues and has been shown to be highly efficacious for various somatic and psychiatric disorders [25, 26], including OCD [27–32]. ICBT for OCD has also proven effective in routine care [32]. Therefore, ICBT can overcome several treatment barriers and provide OCD patients with an evidence-based therapy (e.g. [33–37]. Internet-delivered interventions, compared to face-to-face (f2f) treatment, offer the convenience of immediate and comfortable access to treatment and allow patients to manage their own pace and preferences as they process through the program. ICBT interventions can be offered as a therapist-guided or self-guided programs. In therapist-guided interventions, patients go through the written self-help materials and homework assignments while receiving support and encouragement from an identified therapist via chat or email messages. Differently, self-guided interventions do not involve clinician or therapist support during the treatment [36]. These programs have been shown to be effective [36, 38–41], although most recent studies regarding guided ICBT programs indicate a superiority of guided interventions over self-help [27, 41–45]. However, data from a recent comprehensive literature review suggest that there are no significant efficacy differences between guided and unguided self-help ICBT programs in managing OCD symptoms [46].

The OCD-NET ICBT program was developed in Sweden and has been evaluated in the US and Great Britain over the past decade[27, 31, 47–51]. As with regular CBT for OCD, the main treatment component is ERP [52]. The OCD-NET program led to significant improvements in OCD symptoms (assessed via the same outcome measures) in previous studies [27, 51]with 60% of participants showing clinically significant improvement (95% CI 46–72) in the ICBT group compared to 6% (95% CI 1–17) in the control condition [27]. Clinically significant improvement had been determined here by the Jacobson & Truax- criteria [53], where patients (a) made a statistically reliable baseline to post-treatment improvement and (b) obtained a post-treatment score 2 standard deviations below the mean pre-treatment value. These results were sustained at follow-up assessments. In the trial evaluating OCD-NET in Great Britain, the intervention was associated with large reductions in self-reported OCD symptoms (*d* = 1.77), anxiety (*d* = 1.55), and depression (*d* = 0.8), which were on par with face-to-face CBT for OCD in the same healthcare context [31]. Additionally, the majority of participants were satisfied with the online treatment and found the online materials helpful [31].

The aim of this study was to investigate the acceptability, feasibility, and effectiveness of the ICBT program for OCD, designed as a naturalistic pilot trial during the COVID-19 pandemic in a psychiatric outpatient department in Germany.

## Material and methods

### Study design

This naturalistic, longitudinal, prospective intervention study evaluates the acceptability, feasibility, and effectiveness of ICBT for OCD (OCD-NET) during the COVID-19 pandemic in a psychiatric outpatient department in Germany.

### Participants

Recruitment took place between April 2020 and March 2022. This time frame encompasses the range of pandemic restrictions in Germany [54, 55]: from several weeks in hard lockdowns (Spring 2020 and Winter 2020/2021, including school closures, closed borders, mask mandates, closed hospitals and nursing homes, etc.), partial lockdowns (with various restrictions on meetings and recreational activities, and partial reopening of schools and offices), to the loosening of restrictions in line with vaccination campaigns (started in December 2020); and finally to no major restrictions for daily life, though some measures (e.g., face masks) remained in place for the healthcare sector and hospitals (March/April 2022). Due to the pandemic restrictions and disorder-related fears of infection and contamination [19, 20, 24, 41], several patients with OCD did not have access to the treatment facilities of the specialized outpatient department for several weeks. This study was initially set up under urgent procedures and included several flexible adaptations to respond to the evolving pandemic situation (e.g., different methods of obtaining informed consent, flexibility in terms of blended care i.e., f2f appointments).

This study was conducted in accordance with the principles of the Declaration of Helsinki. Approval was granted by the Ethics Committee of the University of Leipzig, Medical Faculty (31.03.2020, file reference: 136–20/ek, SARS-CoV-2 urgent procedure). Informed consent was obtained from all individual participants included in the study. Consent was initially obtained via telephone interview (due to lockdown measures) with the guiding therapist in the beginning of the recruitment process, and later via written informed consent from each participant.

All patients 18 years or older with a primary diagnosis of OCD and already in (f2f) treatment in the OCD outpatient department, and who had sufficient German language skills, reading and writing ability, and internet access were considered eligible and could be included in the trial. There were no exclusion criteria. According to OCD treatment guidelines, medication with SSRIs is also offered to patients as a combination therapy. The OCD diagnosis was initially assigned during a standardized diagnostic procedure in the specialized OCD outpatient department (from where all participants were recruited) by psychiatrists in training and licensed clinical psychologists (including clinical interviews and standardized self-report measures).

The recruitment process was dynamic and had to be adapted closely to the changing conditions of the pandemic (as well as to the changing availability of f2f treatment services in the outpatient clinic). All participants were already formal patients in the OCD outpatient clinic and had at least completed the diagnostic step mentioned above. Very few participants (*n* = 5) have already attended f2f appointments with the psychiatrist and/or psychotherapist before the pandemic. All other patients in this study were recruited during (changing) pandemic conditions. This included periods where patients could (due to governmental restrictions) only use the OCD-NET program and telephone appointments with the treatment team, as well as periods when patients could use the OCD-NET program and regular f2f appointments with the treatment team. In total, *N* = 57 patients with OCD were invited and recruited for this pilot trial and gave their written informed consent. None refused to participate. After completing (i.e., reading the main text) at least four out of 10 modules of the OCD-NET program (corresponding to a minimum of four weeks of treatment), participants were defined as completers (see [31, 51] for further reference), since they had reached the core intervention exposure with response prevention. Participants who completed up to three modules of the program were defined as non-completers. Participants who never logged into the platform were considered dropouts.

### OCD-NET program

OCD-NET is an evidence-based, therapist-guided, internet-delivered intervention for OCD [27, 31, 48, 49, 51]. Participants log onto a secure website and engage in self-help resources in the form of 10 modules. Each module ends with a homework assignment. The therapist closely and frequently monitors the activities (modules and homework) by providing asynchronous written feedback via the platform. The modules themselves consist of text materials and worksheets. Modules 1–4 cover psychoeducation, cognitive restructuring of metacognitions, and an introduction to ERP. In modules 5–10, the focus is mainly on ERP exercises, which the patient is encouraged to practice daily. The patient is given access to the internet platform with instructions to complete one module per week but is free to progress through the treatment at his or her own pace.

All therapists were licensed psychologists/counselors with extensive experience in treating OCD. Therapists responded to messages from patients within 24 h on weekdays. If a patient had been inactive for 3–4 days, the therapist contacted the patient to help resolve potential issues. The program (all modules and the platform itself) was translated from English to German by medically and therapeutically experienced staff using the back-translation method on random text samples. All therapists received a workshop and hands-on training from experienced ICBT therapists and OCD-NET developers prior to the pilot study. For further information regarding the intervention, please refer to previous descriptions [27, 49].

### Measures

Within the OCD-NET program, participants completed online self-report questionnaires at baseline, weekly during treatment, and at post-treatment (i.e., directly after the end of the treatment), which were administered through the program’s online platform.

### Sociodemographic information

At baseline, information regarding participants’ age, gender, relationship status, educational background, occupational background, and current sick leave was collected. Additionally, the presence of comorbid mental disorders, years with significant OCD symptoms, previous experience with CBT in the treatment of OCD, and current medication usage were assessed. Participants were also asked about their main reason for participation in OCD-NET.

### Primary outcome measure

Feasibility and acceptance of the OCD-NET program was assessed via recruitment and retention rate, adherence and patient engagement and user satisfaction:

#### Satisfaction and engagement

At post-treatment, participants were asked to complete the Client Satisfaction Questionnaire-8 (CSQ-8) [56, 57] to measure their satisfaction with OCD-NET. All items were measured on 4-point Likert scales from 1 = lowest satisfaction to 4 = highest satisfaction. The total sum scores ranged from 8 to 32, with higher scores indicating greater satisfaction. The internal consistency of the questionnaire is high, with Cronbach’s alpha = 0.88.

Participant engagement was measured by module completion, as well as the number of messages sent and received by participants.

#### Questionnaire completion

Of the *N* = 57 participants, *n* = 56 (98.2%) completed the baseline questionnaires, and *n* = 35 (61.4%) completed questionnaires at post-treatment. The completion rates for weekly questionnaires (OCI-R, PHQ-9) ranged from *n* = 29 (50.9%, at week 10) to *n* = 45 (78.9%, at week 2).

### Secondary outcome measures

The secondary outcome measure was the Obsessive–Compulsive Inventory—Revised (OCI-R). Due to pandemic circumstances, this self-report measure was chosen instead of a clinical interview. It assesses various domains of OCD symptoms on 18 items [58]. Items are scored from 0 = “not at all” to 4 = “extremely” on a 5-point Likert scale, resulting in a total sum score ranging from 0 to 72. The OCI-R is a well-established instrument with excellent psychometric properties [58, 59]. The internal consistency of the questionnaire is high, with Cronbach’s alpha = 0.85. The questionnaire was administered at baseline, weekly during the intervention, and at post-treatment.

#### Response and remission

Response and remission at post-treatment were defined based on OCI-R scores, in accordance with recently published criteria [60]: The cut-off for treatment response was a ≥ 40% reduction on the OCI-R, while for remission status was ≤ 8 points.

#### Depressive symptoms

Depressive symptoms were assessed with the Patient Health Questionnaire-9 (PHQ-9) [61]. Symptoms were rated on a 4-point Likert scale, ranging from 0 = “not at all” to 3 = “nearly every day,” to assess the occurrence of depressive symptoms over a two-week period. A higher total sum score (range: 0–27) indicates more severe depressive symptoms. Scores of 10 or more suggest clinically relevant symptoms [62]. The internal consistency of the questionnaire is high, with Cronbach’s alpha = 0.87.The PHQ-9 was administered at baseline, weekly during the intervention, and at post-treatment.

#### Quality of life

At baseline and at post-treatment, participants’ quality of life was assessed using the World Health Organization Quality of Life assessment (WHOQOL-BREF) [63]. The assessment covers different domains of quality of life: physical, psychological, social, and environmental quality of life via 26 items. Items were rated on a 5-point Likert scale from 1 = “not at all” to 5 = “extremely.” For the four domains of quality of life, an index score was calculated, ranging from 0 to 100, with higher index scores indicating higher quality of life. The internal consistency of the questionnaire is high, with Cronbach’s alpha = 0.84.

#### Credibility and expectancy

To measure credibility and expectancy related to the intervention, the Credibility Expectancy Questionnaire (CEQ) [64] was administered at baseline and at post-treatment. The wording of the items was adapted to the OCD-NET program if necessary. The CEQ consists of six items in total, with three items measuring a credibility factor and three items measuring an expectancy factor. The three items of the credibility factor, as well as item 2 of the expectancy factor, were rated on a scale from 1 = lowest expectancy to 9 = highest expectancy, while items 1 and 3 of the expectancy factor were rated on an 11-point scale from 0% = lowest credibility to 100% = highest credibility. The latter scales were transformed into scales from 1 to 9 to compute sum scores, resulting in two sum scores for credibility and expectancy, respectively, with a range from 3 to 27 for each score. Higher sum scores represent higher credibility or expectancy. The internal consistency of the questionnaire is acceptable, with Cronbach’s alpha = 0.66.

#### Self-efficacy

The General Self-Efficacy Scale (GSES) [65] assesses the general sense of perceived self-efficacy with ten items on a 4-point Likert scale from 1 = “not true at all” to 4 = “exactly true.” Items are added to a sum score ranging from 10 to 40. Higher values indicate higher self-efficacy. The GSES was administered at baseline and post-treatment. The internal consistency of the questionnaire is high, with Cronbach’s alpha = 0.87.

#### Psychological problems and symptoms of psychopathology

The Mini-Symptom-Checklist (Mini-SCL) measures psychological distress over the past seven days. A Global Scale Index (GSI) was computed as the mean score of all 18 items, which were rated on 5-point Likert scales, ranging from 1 = “not at all” to 5 = “very much.” Higher GSI scores indicate higher impairment. The Mini-SCL was administered at baseline and post-treatment. The internal consistency of the questionnaire is high, with Cronbach’s alpha = 0.84.

#### Therapeutic alliance

The Working Alliance Inventory Short-Revised (WAI-SR) [66, 67] measures therapeutic alliance on 12 items on a 5-point Likert scale from 1 = “seldom” to 5 = “always.” Sum scores range from 12 to 60, with higher scores indicating stronger working alliance. The scale was administered at week 3 of the treatment. The internal consistency of the questionnaire is high, with Cronbach’s alpha = 0.89.

### Statistical analysis

Statistical analyses were performed using R (version 4.3.2; [68] and SPSS (version 29.0; [69]. The two-sided level of significance was α = 0.05.

The OCI-R and PHQ-9 data, assessed at baseline, weekly, and post-treatment, were analyzed using linear mixed-effects models with the *lme4* R-package [70]. The models included a random intercept for each participant and fixed effects for time. The models were fitted using maximum likelihood estimation. Standardized effect sizes were calculated based on the least-squares means using the residual standard deviation of the random effects as sigma.

All other outcome measures (WHOQOL-BREF, CEQ, GSES, Mini-SCL), which were assessed at baseline and post-treatment, were analyzed using paired *t*-tests in SPSS. Bonferroni correction was applied for multiple testing. For effect size estimations, Cohen’s *d* was interpreted as small, *d* = 0.2; medium, *d* = 0.5; and large, *d* = 0.8 [71].

Missing data was imputed using chained random forests in the *missRanger* R-package [72]. First, all analyses were conducted according to intention to treat (ITT,* n* = 57). Second, sensitivity analyses included only completers (*n* = 45).

## Results

### Participant characteristics

The majority of participants were female (*n* = 33, 58.9%), with a mean age of 33.05 (*SD* = 10.75, range 19–65) years. They reported significant OCD symptoms for an average of 12.70 years (see Table 1 for further details). In total, *n* = 29 (51.8%) participants reported diagnoses of one or more comorbid mental disorders (see Table 1). Additionally, *n* = 42 (75.0%) participants reported current use of psychotropic medication (SSRIs).

**Table 1:** Participant characteristics (*n* = 57)

	*n* (%)
Female gender	33 (58.9)
Age, *M* (*SD*)	33.05 (10.75)
Relationship status	
Not married	41 (73.2)
Married	12 (21.4)
Divorced	3 (5.4)
Education (≥ 12 years)	40 (71.4)
Occupational status	
Working full-time	26 (46.4)
Working part-time	12 (21.4)
Student	10 (17.9)
Retired	1 (1.8)
Unemployed	2 (3.6)
Disability pension	5 (8.9)
Currently on sick leave	6 (10.7)
Years with OCD, *M* (*SD*)	12.70 (10.55)
Previous CBT for OCD	27 (48.2)
Presence of comorbid mental disorder	29 (51.8)
Comorbid mental disorder diagnosis^a^	
Unipolar depression	20 (35.7)
Anxiety disorder	10 (17.9)
Personality disorder	5 (8.9)
ADD/ADHD	2 (3.6)
Other^b^	5 (8.9)
Current medication	42 (75.0)

*M* mean, *SD* standard deviation, ADD/ADHD attention deficit disorder/attention deficit hyperactivity disorder

^a^Multiple answers were possible

^b^Other diagnoses include: anorexia nervosa, somatoform disorder, substance use disorder

### Reasons for participation in OCD-NET

Thirteen participants (23.2%) reported that their main reason for participating in the program was that their OCD symptoms had worsened during the pandemic. Twelve participants (21.4%) reported that they participated in the program due to pandemic-related restrictions in (psycho)therapeutic services. Fourteen participants (25.0%) reported that they had enough time to invest in such a program due to the pandemic. Finally, *n* = 17 (30.4%) participants reported other reasons for participation, including a general worsening of symptoms or a desire to try an online program due to geographic or work schedule restrictions (e.g.,., shift work), which made it difficult for them to attend regular face-to-face therapy.

### Primary outcome: module completion

**Fig. 1:**
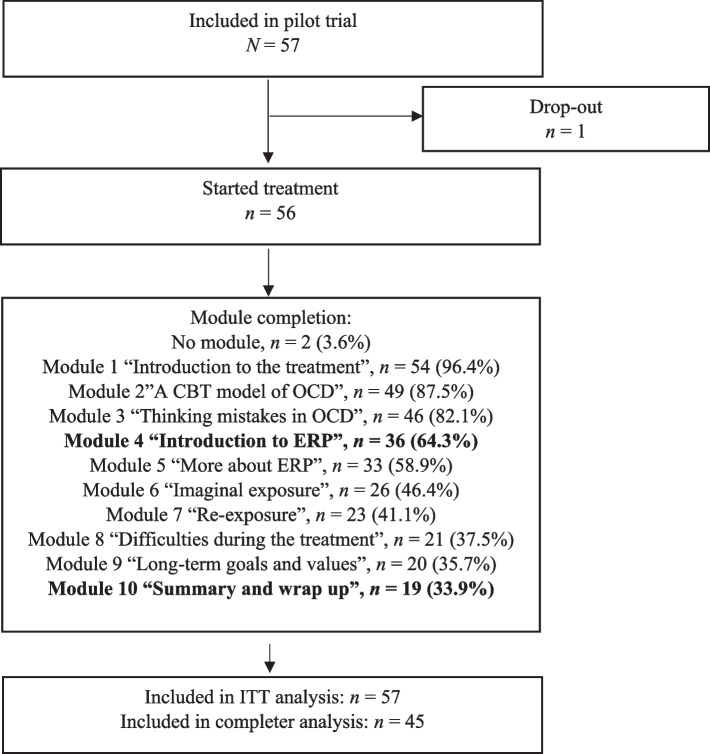
Participation flowchart

Of the *N* = 57 participants, *n* = 45 (78.9%) were considered treatment completers, *n* = 11 (19.3%) were non-completers, and *n* = 1 (1.8%) never logged in and was considered a dropout. On average, participants completed 6.30 (*SD* = 3.21) modules, and *n* = 19 (33.9%) participants completed all 10 modules of the program (see Fig. 1 for details on all modules).

### Primary outcome: satisfaction, and engagement

The CSQ-8 had a post-treatment mean sum score of 24.05 (*SD* = 4.35) in the ITT sample and 24.47 (*SD* = 4.19) in the completer sample. Most participants reported that they would generally or definitely recommend OCD-NET to a friend in need of similar help (ITT: *n* = 56, 98.2%; completer: *n* = 44, 97.8%), and that the program provided the support they needed (ITT: *n* = 49, 86.0%; completer: *n* = 41, 91.1%). On average, participants sent 4.21 (*SD* = 4.55, range 0 – 19) messages and received 9.40 (*SD* = 4.77, range 1 – 23) messages from their therapist.

### Secondary outcome (OCI-R)

In the ITT analyses (*n* = 57), the OCI-R decreased from 26.10 at baseline to 20.67 at post-treatment (mean reduction = − 5.43 points), with a within-group effect size of *d* = 1.13 (95% CI 0.88 – 1.38; see Table 2 and Figure 2 (supplementary material) for detailed results). This reduction was statistically significant, *F*(1, 627) = 86.28, *p* < 0.001. Sensitivity analyses were performed on the *n* = 45 completers. The estimated pre–post change was also significant for treatment completers, with a mean reduction of –5.44 points, *d* = 1.21 (95% CI 0.93—1.50), *F*(1, 495) = 78.36, *p* < 0.001). The Little test for missing data on the OCI-R was not statistically significant (*n* = 57, χ^2^ = 331.78, *p* = 0.39), supporting the assumption that data were missing at random.

**Table 2:** Estimated means and 95% confidence intervals for the OCI-R (*n* = 57; ITT analysis)

Assessment (week)	*M*	*SE*	df	95% Confidence Interval	
				Lower limit	Upper limit
0 (Baseline)	26.10	1.76	61.43	22.59	29.61
1	25.61	1.75	60.29	22.11	29.10
2	25.11	1.74	59.39	21.63	28.59
3	24.62	1.74	58.72	21.14	28.09
4	24.12	1.73	58.27	20.66	27.59
5	23.63	1.73	58.05	20.17	27.09
6	23.14	1.73	58.05	19.67	26.60
7	22.64	1.73	58.27	19.17	26.11
8	22.15	1.74	58.72	18.67	25.62
9	21.65	1.74	59.39	18.17	25.14
10	21.16	1.75	60.29	17.67	24.66
11 (Post-treatment)	20.67	1.76	61.43	17.16	24.18

### Secondary outcome (PHQ-9)

In the ITT analyses (*n* = 57), the PHQ-9 decreased from 10.80 at baseline to 8.16 at post-treatment (mean reduction = − 2.64 points), with a within-group effect size of *d* = 0.90 (95% CI 0.65 – 1.15; see Table 3 and Figure 3 (supplementary material) for detailed results). This reduction was statistically significant, *F*(1, 627) = 54.34*, p* < 0.001. In the completer analysis, the estimated pre–post change was also significant (mean reduction = –2.68 points, *d* = 0.90 [95% CI 0.62—1.18], *F* [1, 495] = 43.40, *p* < 0.001).

**Table 3:** Estimated means and 95% confidence intervals for the PHQ-9 (*n* = 57)

Assessment (week)	*M*	*SE*	df	95% Confidence Interval	
				Lower limit	Upper limit
0 (Baseline)	10.80	0.70	66.45	9.40	12.20
1	10.56	0.69	63.61	9.17	11.95
2	10.32	0.69	61.37	8.94	11.70
3	10.08	0.68	59.72	8.71	11.45
4	9.84	0.68	58.63	8.48	11.20
5	9.60	0.68	58.09	8.24	10.96
6	9.36	0.68	58.09	8.00	10.72
7	9.12	0.68	58.63	7.76	10.48
8	8.88	0.68	59.72	7.51	10.25
9	8.64	0.69	61.37	7.27	10.02
10	8.40	0.69	63.61	7.02	9.79
11 (Post-treatment)	8.16	0.70	66.45	6.76	9.57

### Secondary outcomes (WHOQOL-BREF, CEQ, GSES, Mini-SCL, WAI-SR)

Regarding the WHOQOL-BREF, no significant differences were found between baseline and post-treatment scores in any of the four subscales, either in ITT or completer analyses (all *p* > 0.05, see Table 4).

**Table 4:** Pre-post comparison of secondary outcome measures (*n* = 57)

	Baseline *M (SD)*	Post-treatment *M (SD)*	Test result	*p*	*d* [95% CI]
Quality of life (WHOQOL-BREF)					
Physical	14.63 (2.67)	14.40 (3.05)	*t*(56) = 0.83	.410	0.11 [−0.15; 0.37]
Psychological	12.32 (2.83)	12.95 (3.12)	*t*(56) = −1.87	.067	−0.25 [−0.51; 0.02]
Social	13.05 (3.49)	13.05 (3.67)	*t*(56) = 0.00	.999	0.00 [−0.26; 0.26]
Environmental	15.75 (1.94)	15.39 (2.56)	*t*(56) = 1.58	.120	0.21 [−0.05; 0.47]
Treatment credibility and expectancy (CEQ)					
Credibility	19.47 (4.48)	19.42 (4.43)	*t*(56) = 0.09	.929	0.01 [−0.25; 0.27]
Expectancy	12.95 (5.07)	10.65 (4.81)	*t*(56) = 3.44	**.001**	0.46 [0.18; 0.73]
Self-efficacy (GSES)	25.26 (5.13)	26.39 (5.69)	*t*(56) = −2.02	**.048**	−0.27 [−0.53; −0.00]
Psychological distress (Mini-SCL; GSI)	0.85 (0.48)	0.87 (0.61)	*t*(56) = −0.28	.782	−0.04 [−0.30; 0.22]

*M* mean, *SD* standard deviation, *WHOQOL-BREF* World Health Organization Quality of Life assessment, *CEQ* Credibility Expectancy Questionnaire, *GSES* General Self-Efficacy Scale, *Mini-SCL* Mini-Symptom-Checklist, *GSI* Global Scale Index

Regarding the CEQ, treatment credibility showed no significant difference between baseline and post-treatment scores in either ITT or completer analyses (all *p* > 0.05; small effects). However, expectancy decreased slightly and significantly from baseline to post-treatment in both analyses (all *p* < 0.01). Overall, participants reported high levels of treatment credibility and expectancy of improvement at baseline (see Table 4).

Self-efficacy as assessed using the GSES, increased significantly from baseline to post-treatment in both the ITT and completer analyses (all *p* < 0.05; small to medium effects). In contrast, Mini-SCL GSI scores showed no significant differences in either analyses (all *p* > 0.05).The WAI-SR mean sum score was 42.19 (*SD* = 9.21) in the ITT sample (completer sample: *M* = 43.11, *SD* = 9.52).

### Response and remission

Among all participants, *n* = 17 (29.8%) showed a treatment response on the OCI-R, and *n* = 9 (15.8%) met the criteria for remission. Among the *n* = 45 completers, the rates of treatment response (*n* = 13, 28.9%) and remission (*n* = 8, 17.8%) were comparable.

## Discussion

The aim of this study was to evaluate the acceptability, feasibility, and effectiveness of the ICBT program “OCD-NET” for patients with OCD in Germany. The OCD-NET program is overall highly acceptable and appears to meet patients’ needs in routine care, even under pandemic constraints.

Methods for evaluating treatment satisfaction have varied between studies, but the findings from the current study (86% felt the treatment provided the support they needed) are in line with previous findings of high rates of satisfaction with the online treatment. Although these results are encouraging, not all patients respond to treatment and future research should examine how to further improve clinical outcomes [30], for example by identifying patient or treatment characteristics that moderate outcomes [73].

Recruitment for the current study took place during the COVID-19 pandemic (April 2020 to March 2022), which may have influenced the outcomes in multiple ways. First, patient motivation and engagement with treatment may have been higher than usual, as access to face-to-face treatment was limited due to lockdown measures in Germany during this period. A study from Australia, for example, reported a 500% increase in uptake of ICBT for OCD from March to October 2020 compared to the previous year [74]. Second, a clinical observation from the outpatient department in the beginning of the COVID-19 pandemic noted that many patients with OCD reported feeling overall less ill, as the general population started washing and disinfecting their hands more frequently. This is in line with early reports showing that symptom relief occurred among some OCD patients, and that the majority reported no impact of the pandemic on their OCD symptoms [75]. Overall, the literature shows that patients with OCD experienced a worsening of symptoms of OCD during the COVID-19 pandemic, but responses were heterogeneous [76].

The treatment resulted in statistically significant and clinically relevant reductions in OCD symptoms, with mean reductions on the OCI-R of − 5.43 and –5.44 points in ITT and completer analyses, respectively. Around 29% of participants showed a treatment response on the OCI-R (a reduction of ≥ 40% from baseline), while 16% and 18% in the ITT and completer analysis, respectively, met criteria for remission (an OCI-R score of 8 or lower). In addition, overall treatment satisfaction was high and treatment credibility and expectancy were also comparable to those observed in recent studies of online interventions for psychiatric outpatients [77]. The results of this pilot study add to the emerging literature of ICBT for OCD implementations in real-world healthcare settings, showing that the treatment can be delivered safely with clinically significant improvements [31, 48, 51, 78]. For example, the within-group effect size in self-rated OCD symptoms on the OCI-R (d = 1.13) was higher compared to a previous evaluation in the US (d = 0.92) [78] but lower compared to evaluations of OCD-NET in Sweden (d = 2.12) [51] and the United Kingdom (d = 1.77) [31]. Treatment completion rates (78%) were comparable to the Swedish (87%, [51]) and US (70%, [78]) studies, and higher than in the study in the United Kingdom (55%, [31]).

Similarly, improvements on secondary outcomes such as depressive symptoms (*d* = 0.9) were in the sense of previous studies (e.g., *d* = 0.8 in the study in the United Kingdom) [31].

Another notable finding is that self-efficacy significantly increased from baseline to post-treatment, which is expected from an effective online self-management program. This suggests that the ICBT program can help patients feel more confident in their ability to manage their condition.

The participants in this study reported good working alliance with their guiding therapists. The scores are similar to other ICBT programs also [79, 80], however suitable normative data for the interpretation of the WAI-SR are not available.

### Strengths and limitations

A strength of the study lies in its naturalistic design with broad inclusion criteria; therefore it was possible to study a representative sample of the population of adults with OCD seeking outpatient psychiatric treatment. For example, medication with SSRIs (according to the treatment guidelines for OCD) was reported by 75% of the patients, which is higher than in similar studies [27, 31]. Another strength is the high completion rate (78%), which is higher than in several previous studies on ICBT for OCD [30, 81–83].

Nonetheless, some limitations should be mentioned. First, the sample size was relatively small, which may have reduced study’s power to detect small- to medium-sized effects in some clinical outcomes of interest. Second, there were no clinician-assessed outcomes of OCD symptoms (e.g., the Yale-Brown Obsessive–Compulsive Scale) or diagnostic interviews conducted after treatment, which might have indicated different results from the OCI-R findings as the clinician-rated assessments address overall severity of OCD rather than specific symptoms. Additionally, no follow-up assessments were conducted to evaluate the long-term effects. Furthermore, a key limitation of the study is the absence of a control condition, which prevents the assessment of regression to the mean, particularly in relation to increased symptom severity due to the pandemic. Specifically, participants may have experienced stress related to the pandemic, and any symptom improvement could be attributed to a natural adjustment process over time, rather than the intervention itself.

An important next step for this line of research is to conduct a sufficiently powered randomized controlled trial employing gold-standard assessments of OCD symptoms and comparing the intervention to an active or waitlist control condition.

## Conclusion

Improvements were observed in self-rated measures of OCD and depression. Overall, the OCD-NET program is highly acceptable and appears to meet patients’ needs in routine care, even under pandemic conditions. It can be concluded that OCD-NET is feasible, acceptable and provides an effective treatment option for individuals with OCD under pandemic and non-pandemic conditions. The results also suggest that this ICBT program can be integrated into routine psychiatric outpatient treatments. However, future research should investigate how to achieve effective upscaling and sustainable implementation.

## Supplementary Information

Supplementary Material 1: Figure 2. Estimated means and 95% confidence intervals for OCI-R (*n* = 57). Note. Week 0 corresponds to baseline assessment, week 11 to post-treatment assessment. Figure 3. Estimated means and 95% confidence intervals for PHQ-9 (*n* = 57). Note. Week 0 corresponds to baseline assessment, week 11 to post-treatment assessment.

## Data Availability

All relevant data files are available from the OSF database, Identifier: DOI 10.17605/OSF.IO/6YSZG.
