# Supplementary material for: Acceptability, feasibility, and effectiveness of internet-based cognitive behavior therapy for obsessive–compulsive disorder (OCD-NET): a naturalistic pilot trial during the COVID-19 pandemic in a psychiatric outpatient department in Germany
Source: BMC Psychiatry. 2025 Jan 30;25:85. doi: 10.1186/s12888-025-06519-7 (PMC11783832; doi:10.1186/s12888-025-06519-7)
Supplement: Supplementary file 1 — Supplementary Material 1: Figure 2. Estimated means and 95% confidence intervals for OCI-R (n = 57). Note. Week 0 corresponds to baseline assessment, week 11 to post-treatment assessment. Figure 3. Estimated means and 95% confidence intervals for PHQ-9 (n = 57). Note. Week 0 corresponds to baseline assessment, week 11 to post-treatment assessment. [file 12888_2025_6519_MOESM1_ESM.docx]

**Supplemetary material for:**

Acceptability, feasibility, and effectiveness of iInternet-based cognitive behaviour therapy for obsessive-compulsive disorder (OCD-NET): A Nnaturalistic pilot trial during the COVID-19 pandemic in a psychiatric outpatient department in Germany


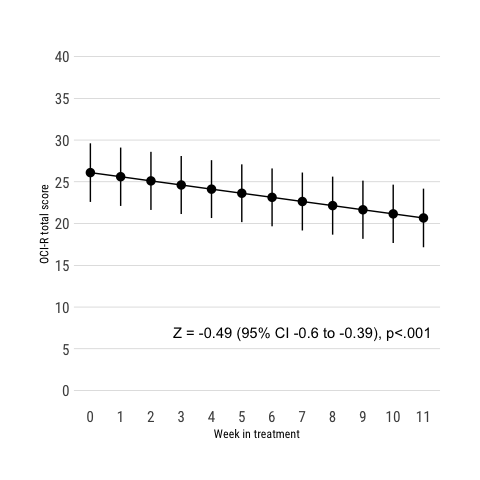


*Figure 2.* Estimated means and 95% confidence intervals for OCI-R (*n* = 57).

*Note.* Week 0 corresponds to baseline assessment, week 11 to post-treatment assessment.


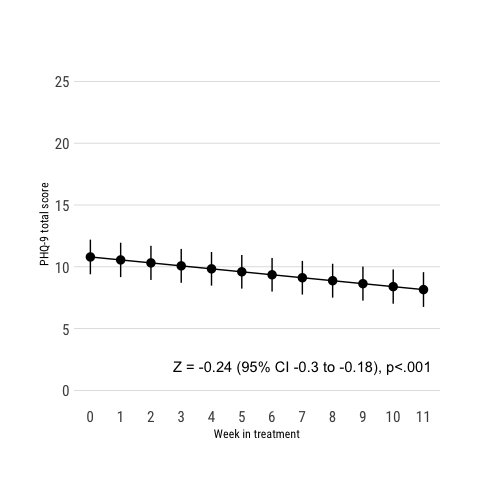


*Figure 3.* Estimated means and 95% confidence intervals for PHQ-9 (*n* = 57).

*Note.* Week 0 corresponds to baseline assessment, week 11 to post-treatment assessment.
